# Rabies in Sri Lanka: Splendid Isolation

**DOI:** 10.3201/eid0903.020545

**Published:** 2003-03

**Authors:** Susilakanthi Nanayakkara, Jean S. Smith, Charles E. Rupprecht

**Affiliations:** * Centers for Disease Control and Prevention, Atlanta, Georgia, USA; † Medical Research Institute, Colombo, Sri Lanka

**Keywords:** rabies virus, Sri Lanka, dogs, communicable disease control, lyssavirus, virus typing, phylogenetic analysis, dispatch

## Abstract

Rabies virus exists in dogs on Sri Lanka as a single, minimally divergent lineage only distantly related to other rabies virus lineages in Asia. Stable, geographically isolated virus populations are susceptible to local extinction. A fully implemented rabies-control campaign could make Sri Lanka the first Asian country in >30 years to become free of rabies virus.

Rabies, an encephalomyelitis caused by infection with rabies virus or other lyssaviruses, is responsible for 40,000–50,000 human deaths each year in Asian countries (*1*). Human rabies is preventable, but the high cost of antirabies biologics limits their use. Because the source of almost all human rabies infections in Asia is a bite by a domestic dog, effective dog rabies control programs not only serve to reduce human deaths but also can**]** reduce the overall costs associated with rabies prevention.

Sri Lanka is an island with a land area of approximately 62,000 km^2^ situated in the Indian Ocean, 35 km from the southern end of the Indian Peninsula. More than 95% of approximately 100 human rabies deaths each year in Sri Lanka are the result of bites by unvaccinated dogs, and U.S.$1.5 million is spent each year for antirabies biologics (*2*). A national dog rabies elimination program begun 20 years ago has yet to reduce the number of bite exposures requiring rabies treatment or the number of human rabies deaths. Low vaccination coverage in the resident dog population and ineffective management of stray animals are the most likely reasons for the program’s lack of success, but the abundant wild fauna in Sri Lanka could provide unrecognized reservoirs for rabies virus. Serologic data also suggest that lyssaviruses other than rabies virus may be present in bats in Asia (*3*). Additionally, Sri Lanka’s close proximity to India might allow frequent introduction of rabies virus–infected animals. Our research expands an earlier study of rabies in Sri Lanka (*4*) by using a panel of monoclonal antibodies (MAbs) to survey Sri Lankan rabies samples for lyssaviruses other than rabies, by using genetic typing to identify viral lineages that might signify a wildlife reservoir for rabies virus, and by using phylogenetic analysis to obtain estimates of the frequency of introductions of rabies virus from neighboring Asian countries that might jeopardize a dog rabies–control program in Sri Lanka.

## The Study

Most samples submitted for rabies testing in Sri Lanka originate from the area around the capital, Colombo. Our study included 44 samples: 36 from cases chosen randomly from the approximately 180 animals diagnosed with rabies at the Medical Research Institute, Colombo, from January to May 2001; 2 from elephants whose infections in 1998 and 1999 occurred in an area of Colombo with large active bat colonies; and 5 from human case-patients (1999–2001). A dog rabies virus collected in 1986 from Colombo served as a reference sample for mutational change in Sri Lankan rabies virus over time. Sri Lankan samples were submitted to virus typing by both antigenic and genetic methods.

The antigenic profile of 31 Sri Lankan samples, displaying 3+ to 4+ antigen distribution, was determined by indirect immunofluorescence methods with a panel of 20 nucleoprotein-specific MAbs obtained from the Centers for Disease Control and Prevention and the Wistar Institute and compared with published reaction patterns for these MAbs (*5*). Only two antibodies were useful. C4, which is not known to react with any lyssavirus other than rabies virus, reacted with all samples from Sri Lanka, indicating the absence of any other lyssavirus among these samples. A negative reaction with MAb CR54 distinguished rabies virus from Sri Lanka from that from the Philippines. The reaction pattern for Sri Lankan virus was identical to that reported for rabies virus from Indonesia and Thailand.

**Figure 1:**
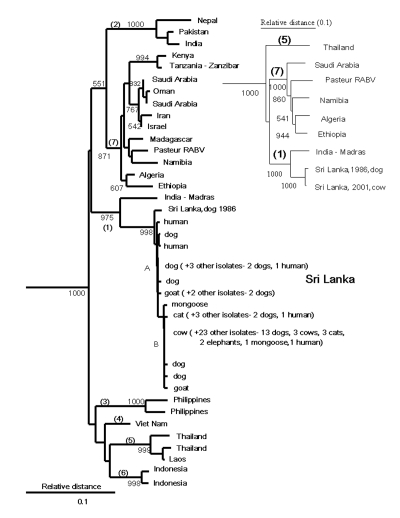
Neighbor-joining tree for 264 base pairs (bp) of nucleoprotein gene sequence (bp 1157–1420) for 44 rabies virus samples from Sri Lanka compared with samples from other areas of Asia and Africa with uncontrolled dog rabies. Samples were aligned with Pasteur rabies virus (GenBank accession no. M13215) by using the PileUp program of the Wisconsin Package version 10 (Genetic Computer Group, 1999, Madison, WI). The phylogenetic analyses were perfomed by using the DNADIST (Kimura two-parameter method), Neighbor (Neighbor-joining method), SEQBOOT, and CONSENSE programs of the PHYLIP package, version 3.5 (available from: URL: http://evolution.genetics.washington.edu/phylip.html). The program TREEVIEW was used to obtain the graphic output (available from: URL: http://taxonomy.zoology.gla.ac.uk/rod/treeview.html). Australian bat lyssavirus (GenBank AF081020) and Duvenhage virus (GenBank U22848) were used as outgroups (not shown). Bootstrap values >50% in 1,000 resamplings of the data are shown at nodes corresponding to the different lineages and sample clusters. The nodes signifying highest order clustering of rabies virus lineages (supported by bootstrap values >70%) are numbered 1–7. The two clusters of Sri Lankan samples, designated by mutations at nucleotides 1231 and 1408, are indicated as A and B. Inset shows a similar tree achieved by analysis of complete nucleoprotein gene sequence (1414 bp). All nonidentical Sri Lankan samples were submitted to GenBank (AY138549–AY138564). The Table lists sample collection information.

A phylogenetic analysis included the 44 Sri Lankan samples and nucleotide sequences for rabies virus and other lyssaviruses obtained from GenBank and from a sequence repository at CDC (Table, Figure 1). Data analyzed included representatives of available sequence for mainland and island Asian countries and sequence data for samples representing a similarly broad geographic distribution of mainland and island countries of East Africa and the Arabian Peninsula. With the use of standard methods (*6*), RNA was extracted from the Sri Lankan rabies virus samples, reverse transcribed, and amplified by polymerase chain reaction. Genetic typing was based on nucleotide sequence differences in complementary DNA (cDNA) for the nucleoprotein gene as aligned with sequence for the Pasteur vaccine strain of rabies virus (*7*).

**Table:** Virus samples from countries other than Sri Lanka included in phylogenetic analysis

Sample ID	Sample history and GenBank accession numbers
Australian bat lysssavirus	*Austr*alian bat lyssavirus from insectivorous bat *(Saccolaimus flaviventris);* 1996. Lyssavirus most closely related to rabies virus. Used as outgroup to root phylogenetic analysis. AF081020.
Duvenhage virus	Duvenhage virus from human bitten by bat, South Africa, 1970. Bat lyssavirus used as outgroup to root phylogenetic analysis. U22848.
Nepal	U.S. citizen bitten by dog in Nepal, 1996. AY138578.
Pakistan	Dog, 1990. AY138565.
India	Resident of U. K., bitten by dog in India, circa 1988. AY138566.
Kenya	U.S. Peace Corps volunteer bitten by dog in Kenya, 1983. AY138567.
Tanzania	Goat, 1992, Zanzibar, Tanzania. AY138568.
Saudi Arabia	Arabian-American Oil Company employee infected in Saudi Arabia, circa 1981, AY138569; fox, 1987. U22481.
Oman	Red fox, 1990. U22480.
Iran	Dog, 1986. U22482.
Israel	Dog, 1993. AY138570.
Madagascar	Human, 1980. AY138571.
Pasteur rabies virus	Cow bitten by dog, Paris, 1882, multiple laboratory passages. M13215.
Namibia	Jackal, 1992. U22649.
Algeria	Dog, 1982. U22643.
Ethiopia	Hyena, 1987. U22637.
India-Madras	Unpublished, 2001. AF374721.
Sri Lanka, reference dog sample, 1986	Dog case sample collected by Dr. Alex Wandeler, Colombo, 1986. AY138549.
Sri Lanka, human cases	Five human case-patients with diagnosed rabies, 1999–2001. Cluster A samples originated from Kandy (n=2) and Ratnapura. Cluster B samples originated from Colombo and Karapitiya. Two cluster A human case samples with nucleotide substitutions not found in consensus sequence were submitted to GenBank. AY138554 and AY138558.
Sri Lanka, dog cases	24 dogs with diagnosed rabies, January–May 2001. Cluster A samples originated from Ragama, Polgasowita, Katunayake, Puttalam, Meerigama, Panadura, Dewulapitiya. Cluster B samples originated from Ragama, Polgasowita, Mirihana, Dehiwela, Moratuwa, Chillaw, Katana, Mount Lavinia, Balapitiya, Wattala (n=2), Payagala (n=2), Colombo (n=3), Nugegoda. Dog samples with nucleotide substitutions not found in consensus sequence for cluster A (AY138553, AY138555, AY138557, AY138559) and cluster B (AY138551, AY138552) were submitted to GenBank.
Sri Lanka, goat cases	Two goats with diagnosed rabies, January–May 2001. Cluster A sample originated from Seeduwa. AY138561. Cluster B sample originated from Kandy. AY138556.
Sri Lanka, mongoose cases	Two mongooses (cluster B) with diagnosed rabies, January–May 2001, Panadura and Moratuwa. Sample with nucleotide substitutions not found in consensus sequence for cluster B was submitted to GenBank. AY138562.
Sri Lanka, cow cases	Four cows (cluster B) with diagnosed rabies, January–May 2001, Kandy, Kaduwela, Horana, Polgasowita. Samples with nucleotide substitutions not found in consensus sequence for cluster were submitted to GenBank. AY138550 and AY138560.
Sri Lanka, elephant cases	Two elephants (cluster B) with diagnosed rabies in 1998 and 1999, in an area of Colombo with large active bat colonies. Sample with nucleotide substitutions not found in consensus sequence for cluster B was submitted to GenBank. AY138564.
Sri Lanka, cat cases	Four cats (cluster B) with diagnosed rabies, January–May 2001, Kelaniya, Nittambuwa, Horana, Kaduwela. Sample with nucleotide substitutions not found in consensus sequence for cluster B was submitted to GenBank. AY138563.
Philippines	Resident of California, 1987, bitten by dog in the Philippines, AY138575; resident of California, 1972, bitten by dog in the Philippines. AY138576.
Viet Nam	Vietnamese refugee in Sidney, Australia, 1991. AY138579.
Thailand	Human, 1983, AY138572; dog, 1995, U22653.
Laos	Laotian immigrant, Texas, 1984. AY138577.
Indonesia	Human, 1989, Jakarta, Java; AY138573. dog, 1989, Java; AY138574.

The 44 samples from Sri Lanka shared approximately 99% homology over a 320-nucleotide region of the carboxy terminus of the N gene (bp 1157–1476). Only 10 nucleotide substitutions (all synonymous) were found when the entire protein encoding sequence of the N gene (1350 bp) was compared for isolates from 1986 and 2001 (1986 dog case no. 1294, GenBank accession no. AY138549; 2001 cow case no. 5657, GenBank accession no. AY138550), and the two samples were identical over an additional 56 bp of nontranslated sequence between the stop codon and polyadenylation signal (bp 1421–1476).

**Figure 2:**
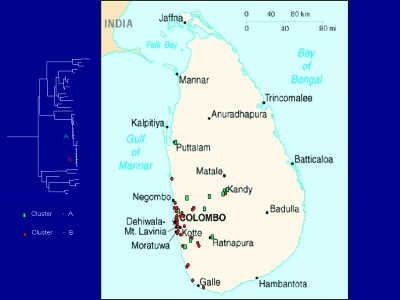
Map of Sri Lanka showing location of rabies samples included in this study. Green, cluster A; red, cluster B.

No evidence was found for a wildlife reservoir for rabies independent of rabies in domestic dogs. Mutations at nucleotide position 1231 and 1408 separated the virus samples into two clusters (Figure 1), but the clusters corresponded roughly to sample collection sites (Figure 2), and domestic dog samples were found in both clusters. Because the analysis included only two mongooses, we cannot exclude mongooses as a cryptic reservoir for rabies. Nevertheless, rabies cases in mongooses and other wild animals are only infrequently identified in Sri Lanka and occur in areas where dog rabies cases are common. Arai et al. (*4*) found similar results for wildlife samples in their study. Given that >95% of human rabies cases report a dog bite as the source of infection, expanding rabies-control programs to include wild animals in Sri Lanka without more specific evidence of a reservoir status for these animals would not be helpful.

In contrast to the limited sequence diversity among samples from Sri Lanka, extensive sequence divergence was found when samples from Sri Lanka were compared with samples from other Asian countries, and genetic change was much more evident in the nontranslated sequence (17.2% to 31.2% divergence) as compared with the protein-encoding sequence (11.8% to 21.5% divergence. Identity for nontranslated sequence in some intercountry comparisons was as low as 50% (e.g., 50% to 58% identity over the nontranslated sequence for comparison of viruses from Indonesia and India). Additionally, samples from Vietnam, Indonesia, Laos, the Philippines, and Thailand contained an extra nucleotide in the nontranslated region as compared with the Pasteur vaccine strain of rabies virus. The exact position of this extra nucleotide could not be determined because it varied in relation to different sample groups, and the single extra nucleotide likely represents multiple insertion and deletion events.

Molecular aspects of the epidemiology of rabies within Sri Lanka and between Sri Lanka and other countries were determined by constructing phylogenetic trees (Figure 1). Because the possibility of multiple insertions and deletions prevented an exact alignment of the nontranslated sequence, phylogenetic analysis was conducted with only the protein-encoding sequence.

The phylogenetic analysis showed that all samples positive for rabies in diagnostic tests from Sri Lanka were rabies virus. Samples from Sri Lanka clustered with the Pasteur vaccine strain of rabies virus and other samples identified as genotype 1 lyssavirus (*8*) and were monophyletic with respect to other lyssavirus genotypes (not shown). Rabies virus samples from Asia were segregated into six clades on the basis of the geographic origin of the samples. Despite the exclusion of the highly variable nontranslated sequence from the analysis, no evidence was found for a common ancestry for all six Asian lineages (Figure 1). Three lineages were composed of samples from a single country (Philippines, Vietnam, and Indonesia). The remaining three lineages comprised samples from geographically proximate countries (Sri Lanka and the sample from Madras, India; Thailand and Laos; and Pakistan, India, and Nepal). Similar findings were achieved with a more limited sample set encompassing the entire nucleoprotein gene (inset in Figure 1).

In contrast to the genetic diversity of Asian rabies virus samples, samples of African 1 rabies virus (*8*) collected from the Arabian Peninsula and mainland and island countries of East Africa shared much higher sequence identity (approximately 93%) and could be resolved as a single lineage sharing a common ancestry with European vaccine strains of the virus dating from the 19th century (Pasteur rabies virus). Molecular clock data suggest that this virus emerged 300–500 years ago (*9*) and support historical data for rabies virus dissemination during early European exploration and colonization of Africa (*8*–*10*). The genetic divergence between samples from Sri Lanka and the most closely related Asian sample (Madras, India; >6.3% difference) suggests a similarly distant common ancestor for these two rabies virus populations.

## Conclusions

As depicted by the antigenic and genetic analysis of samples in this report and the earlier work by Arai et al. (*4*), rabies in Sri Lanka is associated with a single lineage of rabies virus, which shares only a distant common ancestry with rabies viruses from any other Asian country. Genetic diversity between rabies virus lineages from mainland or island Asian countries was much greater than among samples collected over a similar geographic expanse in Africa. Differences between Asia and Africa in the partitioning and degree of spread of rabies virus in host populations might be explained by agricultural practices that evolve in areas of high rainfall versus the agricultural practices necessary in areas where rain is seasonal and scarce. For example, nomadic herding, a common agricultural practice in Africa, would spread an introduced rabies virus over great distances, whereas the primarily small-scale farming operations in Sri Lanka and other Asian countries, maintained locally for generations, could restrict virus spread. These observations suggest that restrictions to domestic animal movement can be exploited in rabies-control efforts in Asia.

Rabies viruses in Sri Lanka and India may be more closely related than described in this and previous studies (*4*). Rabies virus more closely related to Indian viruses may be present in the northern and eastern parts of Sri Lanka, where civil unrest prevented sample collection. The Sri Lankan variant of the virus may still be actively transmitted by animals in India; only limited virus typing has been conducted on Indian rabies samples. However, although considerable genetic diversity was found in the four available sequences for rabies virus from the Indian subcontinent, only one Indian lineage shared a common ancestry with Sri Lankan samples. The degree of divergence between this Indian sample and the Sri Lankan samples and the lack of heterogeneity in Sri Lankan samples collected over a 16-year period suggest that the introduction of the ancestral virus occurred only once and in the distant past.

While the data reported here and in the work by Arai et al. (*4*) represent only a fraction of all rabies cases that occur each year in Sri Lanka, the island is small and heavily populated. If other rabies virus lineages are present, or if introductions from India are more frequent than our data indicate, future virus typing should detect them easily.

Antigenic and genetic typing of samples collected for rabies testing in Sri Lanka found no lyssavirus other than rabies virus, no evidence for cycles of rabies virus transmission in wild species independent of endemic rabies virus in domestic dogs, no evidence of a recent introduction of the virus to Sri Lanka through the translocation of animals from other areas of Asia, and (surprisingly, given the close proximity to mainland Asia) no evidence for outside introduction of the virus to Sri Lanka in the recent past. Historically, rabies of this type has been susceptible to control with a fully implemented vaccination campaign and an animal-control effort that addresses local cultural and religious attitudes toward management of stray animals. The economic impact of rabies on Sri Lanka argues for national and international cooperation in a strong rabies-control program for this island nation. The prospects are good that such an effort could eliminate rabies virus from the island and create the first rabies virus–free country of the millennium.
